# Bubble Relaxation Dynamics in Homopolymer DNA Sequences

**DOI:** 10.3390/molecules28031041

**Published:** 2023-01-20

**Authors:** Malcolm Hillebrand, George Kalosakas, Alan R. Bishop, Charalampos Skokos

**Affiliations:** 1 Nonlinear Dynamics and Chaos Group, Department of Mathematics and Applied Mathematics, University of Cape Town, Rondebosch 7701, South Africa; 2 Department of Materials Science, University of Patras, GR-26504 Rio, Greece; 3 Los Alamos National Laboratory, Los Alamos, NM 87545, USA

**Keywords:** DNA, base pair stretching, bubbles, relaxation, molecular dynamics

## Abstract

Understanding the inherent timescales of large bubbles in DNA is critical to a thorough comprehension of its physicochemical characteristics, as well as their potential role on helix opening and biological function. In this work, we employ the coarse-grained Peyrard–Bishop–Dauxois model of DNA to study relaxation dynamics of large bubbles in homopolymer DNA, using simulations up to the microsecond time scale. By studying energy autocorrelation functions of relatively large bubbles inserted into thermalised DNA molecules, we extract characteristic relaxation times from the equilibration process for both adenine–thymine (AT) and guanine–cytosine (GC) homopolymers. Bubbles of different amplitudes and widths are investigated through extensive statistics and appropriate fittings of their relaxation. Characteristic relaxation times increase with bubble amplitude and width. We show that, within the model, relaxation times are two orders of magnitude longer in GC sequences than in AT sequences. Overall, our results confirm that large bubbles leave a lasting impact on the molecule’s dynamics, for times between 0.5–500 ns depending on the homopolymer type and bubble shape, thus clearly affecting long-time evolutions of the molecule.

## 1. Introduction

The dynamics of biomolecules such as DNA have long been a source of interest, providing meaningful information beyond that yielded by the static molecular structure [1,2,3]. In particular, the notion of extracting timescales for dynamical processes in DNA has attracted attention both theoretically and experimentally [4,5,6,7,8], due to the importance of quantifying the impact of thermal and mechanical effects on the overall behaviour of the molecule.

A particularly interesting feature of DNA dynamics, which has been suggested to have a potential role in transcription and other biological processes, is the existence of local large base pair openings, often called bubbles, where finite regions of the double helix open. These openings can be thermally-induced fluctuations, or promoted by base pair mismatching [9]. DNA bubbles can be experimentally studied through NMR experiments [10] and fluorescence spectra [11,12], as well as computationally using extensive molecular dynamics (MD) simulations [7,13]. It is also possible to study these breather-like excitations using nonlinear modelling (see e.g., [14] and references therein).

There have been many models of DNA proposed and studied in various details [15], ranging from detailed ab initio models [16] to thermodynamically-motivated models [17] and empirical potentials accounting for the helical or curved structure of DNA [18,19,20,21], as well as free-energy-based methods [22]. In this work, we consider the Peyrard–Bishop–Dauxois (PBD) model [23,24,25,26], which provides an effective mesoscale view of DNA dynamics, successfully reproducing sharp denaturation curves and several experimental results [27]. This model reduces the complex molecular structure of the double helix to a more tractable one-dimensional, lattice system, enabling the investigation of such diverse phenomena as intrinsic localised modes [28], electronic transport where bubbles can cause charge trapping [29,30,31], chaoticity [32,33], DNA/TNA couplings [34] and optical switching [35]. The incorporation of a sequence-dependent stacking parameter within the PBD model provides better accuracy with detailed denaturation (i.e., complete separation of the double strand) results for a variety of DNA sequences exhibiting unusual melting behavior [36]. Obviously, due to the coarse-grained character of the model, detailed pathways and intermediate states at the atomic level cannot be addressed.

Using the PBD model, various studies of DNA breathing and fluctuational opening probabilities [37,38,39,40,41] have been carried out. Stretched exponential evolution has been found for the decay of equilibrium fluctuations of base pairs in DNA molecules [42], exhibiting relaxation times beyond the picosecond scale. Opening probability profiles and lifetimes of bubbles have also been studied extensively in the context of DNA promoters [43,44,45,46,47,48,49,50,51,52,53], as well as more general bubble distributions for arbitrary sequences [54]. These findings additionally indicated that more large bubbles can be distinguished in transcriptionally significant regions of DNA promoters than expected from average results, providing additional impetus to the interest of studying longer-time effects of openings in DNA molecules, in which times biological processes might be initiated.

Here, we further investigate bubble lifetimes’ properties by studying relaxation times of large openings in the DNA double strand, such as may be produced by rare thermal fluctuations, induced by artificially engineered means, or naturally created by proteins. Not only does this further the characterisation of DNA’s response to large bubbles, but it provides a quantification of the time scales for the system’s memory of out-of-equilibrium perturbations, and demonstrates that these large openings leave a long-lasting imprint on the dynamics of the molecule.

The paper is organised as follows: In Section 2, we introduce the dynamical PBD model used here, along with the numerical methods, parameters, and the simulation protocols for investigating bubbles. The results and analysis of data follow in Section 3, with the summary and conclusions in Section 4 closing out the report.

## 2. Model and Setup

We perform molecular dynamics simulations using the PBD model of DNA [24], describing the molecule as a sequence of nonlinearly coupled base pairs. The Hamiltonian function of the PBD model for a DNA sequence of *N* base pairs, considering periodic boundary conditions are given by

(1) $$H=\sum_{n=1}^{N}\left[\frac{p_{n}^{2}}{2m}+V_{1}\left(y_{n}\right)+V_{2}\left(y_{n},y_{n-1}\right)\right],$$

with $y_{0}=y_{N}$. The on-site energy interaction is governed by the Morse potential

(2) $$V_{1}\left(y_{n}\right)=D_{n}{\left(e^{-a_{n}y_{n}}-1\right)}^{2},$$

and the nearest-neighbour stacking interaction is modelled by

(3) $$V_{2}\left(y_{n},y_{n-1}\right)=\frac{K_{n,n-1}}{2}\left(1+\rho e^{-b(y_{n}+y_{n-1})}\right){\left(y_{n}-y_{n-1}\right)}^{2}.$$

Here, the $y_{n}$ are the displacements from equilibrium of each base pair, $p_{n}$ the corresponding momenta, $a_{n}$ and $D_{n}$ are the constants of a Morse on-site potential distinguishing AT or GC base pairs, while the coupling constants $K_{n,n-1}$ are sequence-dependent stacking strengths. The parameter values used are $D_{GC}=0.075$ eV, $a_{GC}=6.9$ Å${}^{-1}$ for GC base pairs and $D_{AT}=0.05$ eV, $a_{AT}=4.2$ Å${}^{-1}$ for AT base pairs, ρ=2, b=0.35 Å${}^{-1}$ [27], while, for the $K_{n,n-1}$ values, see Ref. [36] or Table I of Ref. [54]. The parameters of the on-site Morse interaction, Equation (2), for the GC and AT base pairs, as well as the values of ρ and *b* in the stacking potential of Equation (3), have been suggested in Ref. [27] through an accurate description of experimentally obtained melting curves of short DNA molecules by the predictions of the PBD model. These values have been subsequently used in a number of other studies, including the successful comparison between theoretically derived large openings in DNA gene promoters and experimental S1 nuclease cleavage assays [43,44]. The sequence-dependent coupling constants $K_{n,n-1}$ of Equation (3) have been derived later in Ref. [36], by fitting the melting temperatures of homogeneous and periodic DNA chains which exhibit peculiar denaturation transitions. In this work, we consider only the two homopolymer cases, so we have $K_{AA}=K_{TT}=0.0228$ eV/Å${}^{2}$ for pure AT sequences, i.e., poly(dA)·poly(dT) and $K_{GG}=K_{CC}=0.0192$ eV/Å${}^{2}$ for the GC case, poly(dG)·poly(dC). The temperature is kept at a physiological level, around 310K, meaning different energies are used in the AT and GC cases due to our microcanonical constant-energy simulations. Based on the energy-temperature relation in the considered PBD model [54], we use an average energy per particle of $\varepsilon_{i}=0.043$ eV for AT sequences, while for GC sequences $\varepsilon_{i}=0.045$ eV is used.

Importantly, we make use of symplectic integration techniques for the conservative Hamiltonian system [55,56], and specifically the symplectic Runge–Kutta–Nyström method SRKNb6 [57]. Not only is this method efficient and accurate, but it also preserves the system’s symplectic nature, which means that performing simulations even up to several microseconds is possible without sacrificing efficiency for precision, as would be required with typical non-symplectic schemes with growing errors. A relative energy error of around $|H\left(t\right)-H\left(0\right)|/H\left(0\right)<10^{-7}$ is maintained throughout all simulations in this work.

Each simulation is run starting from equilibrium displacements $y_{i}=0$, with a set of initial momenta drawn from a zero-mean random normal distribution, which are scaled to provide the correct total energy as mentioned above. N= 300 base pair long sequences are used, with periodic boundary conditions. This sequence length was selected to ensure that finite size effects are negligible. In addition, we compared results obtained using either periodic or free boundary conditions, finding very similar outcomes at this size, reinforcing that the bubble relaxation dynamics are not significantly affected by the boundaries. For the case of GC homopolymers, 1000 simulations are used to ensure statistical robustness for the results, while, for the AT homopolymers, 2000 simulations are used.

The first 10 ns of the evolution are taken as a thermalisation period, whereafter we directly introduce a bubble by replacing the central base pairs’ displacements with an out-of-equilibrium Gaussian-shaped initial perturbation. This Gaussian has the form

(4) $$y\left(x\right)=h\cdot e^{-\frac{{\left(x-c\right)}^{2}}{2\sigma^{2}}},$$

where *h* is the amplitude of the bubble, *c* is the centre located in the middle of the DNA sequence (i.e., c=150 for our 300-base-pair sequences), and σ the “standard deviation”, characterising the width of the Gaussian. The total number of base pairs which have their displacements replaced by this Gaussian is denoted *w*, which determines the width of the introduced bubble. The parameter σ in Equation (4) is given by σ=w/6 to ensure that the inserted bubble has tails at near equilibrium displacement. Therefore, the inserted out-of-equilibrium bubbles are characterised by their amplitude *h* and width *w*. For all bubbles studied in this work, we keep *w* as an odd number of base pairs to allow for even length tails on either side of the centre. The displacements of the remaining base pairs (those not belonging to the bubble region) are rescaled using a bisection algorithm to retain the total original energy *H* of the chain to within an accuracy of $|H^{\prime }-H|<10^{-10}$ eV, where $H^{\prime }$ denotes the energy of the system after the bubble is inserted and displacements rescaled. With the momenta remaining unaffected, the temperature is unchanged by the bubble insertion.

**Figure 1:**
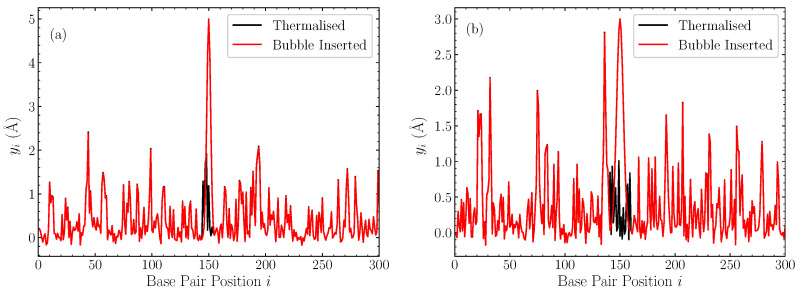
Two representative cases of the displacements in a thermalised homopolymer AT sequence at t=10 ns (black), and the corresponding profile after the Gaussian perturbation is introduced and the remaining displacements rescaled (red). (**a**) bubble with a width w=11 base pairs and amplitude h=5 Å; (**b**) bubble with w=19 base pairs and amplitude h=3 Å.

In Figure 1, we illustrate the profiles of displacements within the DNA chain after the introduction of the bubble, along with the pre-insertion thermalised equilibrium, for two representative cases with bubble width w=11 base pairs and amplitude 5 Å (Figure 1a), and w=19 base pairs and amplitude 3 Å [Figure 1b] in a pure AT sequence. These cases help to put the amplitude and width of these bubbles into context, confirming that these inserted Gaussians are generally out-of-equilibrium large perturbations.

To examine the relaxation of these non-equilibrium bubbles, we track the displacements as the molecule evolves through time, and compute autocorrelation functions for the bubble region. The non-normalised displacement autocorrelation function is calculated as

(5) $$C_{D}\left(t\right)=\frac{1}{w}\langle \sum_{i=(N-w+1)/2}^{i=(N+w-1)/2}y_{i}\left(0\right)y_{i}\left(t\right)\;\rangle ,$$

so the sum over *i* spans the displacements within the region of interest, which is the *w*-base-pair region where the bubble is inserted. The notation 〈·〉 in Equation (5) means that the final autocorrelation function is calculated averaging over the ensemble of 1000 simulations for GC homopolymers and 2000 for AT ones. The limiting or asymptotic value of this correlation function is

(6) $$\chi_{D}=\left(\frac{1}{w}\sum_{i=(N-w+1)/2}^{i=(N+w-1)/2}y_{i}\left(0\right)\right)y_{\mathrm{eq}},$$

where $y_{eq}$ is the average thermal equilibrium displacement of the entire homopolymer sequence. For this value of $y_{eq}$, we use an average of the displacements of all base pairs, from all simulations, after thermalisation and before the bubble is inserted.

In addition to the relaxation of the bubble displacements profile, we also investigate the relaxation of the energy distribution induced by these bubbles. To this end, we compute the local energy at each base pair *i* as

(7) $$\varepsilon_{i}=\frac{p_{i}^{2}}{2m}+V_{1}\left(y_{i}\right)+\frac{1}{2}\left[V_{2}\left(y_{i+1},y_{i}\right)+V_{2}\left(y_{i},y_{i-1}\right)\right],$$

and examine the relaxation of the autocorrelation function for this quantity [42]. For these energies per base pair, $\varepsilon_{i}$ the autocorrelation function is given by

(8) $$C_{E}\left(t\right)=\frac{1}{w}\langle \sum_{i=(N-w+1)/2}^{i=(N+w-1)/2}\varepsilon_{i}\left(0\right)\varepsilon_{i}\left(t\right)\rangle ,$$

where again the sum is over the base pairs inside the bubble region. In the same fashion as for the displacements, the limiting value for the energy autocorrelation function is given as

(9) $$\chi_{E}=\left(\frac{1}{w}\sum_{i=(N-w+1)/2}^{i=(N+w-1)/2}\varepsilon_{i}\left(0\right)\right)\varepsilon_{\mathrm{eq}},$$

where in fact we have explicitly that the equilibrium value of the local energy per base pair is simply the initial energy per base pair. Thus, for GC sequences, $\varepsilon_{eq}^{GC}=0.045$ eV and for AT $\varepsilon_{eq}^{AT}=0.043$ eV.

In order to compute the aforementioned autocorrelation functions in our numerical simulations, the values of the displacements and energies per base pair in the bubble region are recorded starting from the insertion time, which is considered to be t=0. The simulations are run for a further 100 ns for AT sequences, and 5μs for GC sequences, and data stored in log time. These recording times were found to be sufficient for the system to exhibit a complete relaxation of the autocorrelation functions towards equilibrium in the AT case, and very close to this for the GC sequences.

## 3. Results and Discussion

**Figure 2:**
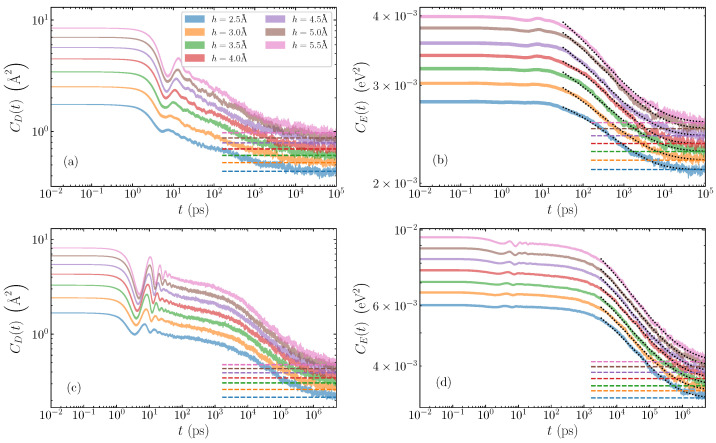
Time evolution of the average autocorrelation functions for homogeneous AT and GC sequences and different initial bubble amplitudes ranging from h=2.5 Å to h=5.5 Å. The shaded region gives the average value plus and minus the standard deviation of the mean. Top row: AT sequences, for a bubble width of w=19 base pairs; (**a**) displacement autocorrelation functions $C_{D}\left(t\right)$, (Equation (5)), and (**b**) energy autocorrelation functions $C_{E}\left(t\right)$ (Equation (8)). Bottom row: GC sequences with a bubble width of w=11 base pairs; (**c**) displacement and (**d**) energy autocorrelation functions. In all cases, the dashed lines mark the expected limiting values $\chi_{D}$ and $\chi_{E}$ from Equations (6) and (9), respectively. Dotted lines in (**b**,**d**) represent stretched exponential fittings with Equation (10).

Illustrative cases of average autocorrelation functions are depicted in log-log scale in Figure 2. For the poly(dA)·poly(dT) sequence (Figure 2a,b), we show the relaxation of a bubble of width w=19 base pairs, and various amplitudes ranging from h=2.5 Å to h=5.5 Å. The displacement autocorrelation functions $C_{D}\left(t\right)$ (Equation (5)) are shown in Figure 2a, and the energy autocorrelation functions $C_{E}\left(t\right)$ (Equation (8)) in Figure 2b. The poly(dG)·poly(dC) case is demonstrated with a bubble of width w=11 base pairs, and the same range of amplitudes h= 2.5–5.5 Å. Figure 2c gives the displacement autocorrelation functions $C_{D}\left(t\right)$, with the energy counterpart $C_{E}\left(t\right)$ seen in Figure 2d. In all plots, for each amplitude, the expected limiting values, provided by Equations (6) and (9) for $C_{D}$ and $C_{E}$, respectively, are indicated by horizontal dashed lines, to which the corresponding autocorrelation functions eventually converge. The shaded regions for each case represent the average autocorrelation function value plus and minus the standard deviation of the mean. The smoothness of these regions (even in log scale), and the small standard deviations, confirm that we have sufficient statistics to eliminate large deviations in the data set.

A typical relaxation process of the AT homopolymers as demonstrated by the displacement autocorrelation function $C_{D}\left(t\right)$ is seen in Figure 2a. Here, we see two distinct stages of the relaxation of the autocorrelation function: an initial oscillatory region until around t=20 ps, with increasing amplitude as the bubble amplitude grows, followed by a steady rapid decay towards the equilibrium value, which is generally reached after several nanoseconds. The energy autocorrelation function $C_{E}\left(t\right)$ (Figure 2b) also exhibits two stages of decay, but the oscillations for the first picoseconds, coinciding with the window of oscillations in the $C_{D}\left(t\right)$ functions, are significantly suppressed. The $C_{E}$ autocorrelations reach the limiting value slightly later than the $C_{D}$ ones.

In the case of GC sequences (Figure 2c,d), similar but distinct decreasing behaviours of the autocorrelation functions are clearly visible. First, there is the initial oscillatory period up to times t= 10–40 ps depending on amplitude, where coherent peaks and troughs are especially visible in the displacement autocorrelation functions (Figure 2c), and less in the large-amplitude energy functions (Figure 2d). In the GC homopolymers, there are more oscillations during the first oscillatory stage of the relaxation in comparison to the AT sequences. When the oscillations diminish, the slow decay of the autocorrelation functions continues up to several nanoseconds in both $C_{D}\left(t\right)$ and $C_{E}\left(t\right)$, which then gives way to the second stage of the rapid decay process lasting until several μs, when equilibrium values are almost reached.

**Figure 3:**
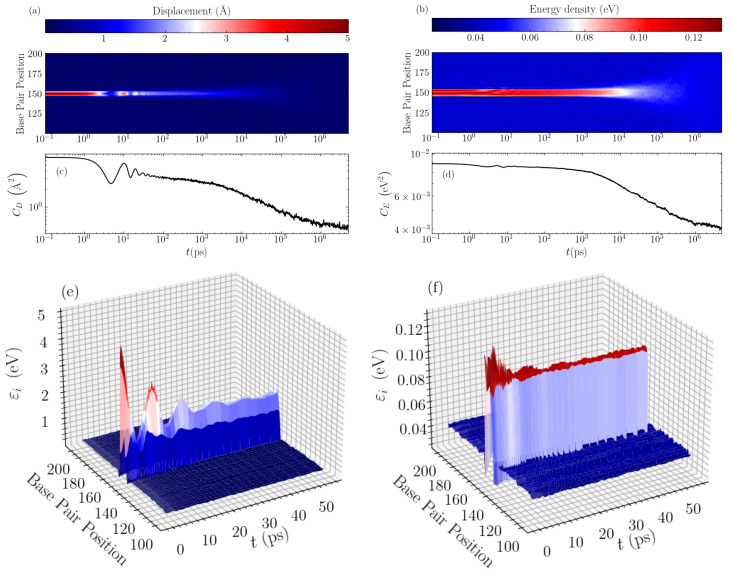
The evolution of the averaged displacement and energy density profiles through time, for the case of a GC homopolymer with an initial bubble of amplitude h=5 Å and width w=11 base pairs. The left column shows data for the displacements and the right column for the energy densities. (**a**,**b**) show a density plot of the long-time evolution profile of the displacements and energy densities, respectively, with the intensity labelled according to the colorbar above the panels. The data are shown for the central 100 base pairs of the sequence. The corresponding average autocorrelation functions, $C_{D}\left(t\right)$ and $C_{E}\left(t\right)$, are presented below, in (**c**,**d**) respectively, for a direct comparison. A 3D representation of the early stage of the evolution is depicted in (**e**,**f**) for the displacements and energy densities, respectively.

In order to find the mechanisms responsible for these distinct relaxations, we directly visualise the time evolution of the average bubble displacements and energy densities. In Figure 3, typical evolution profiles are illustrated for a GC homopolymer with an initial bubble of amplitude h=5 Å and width w=11 base pairs. Considering first the displacement profile shown in a density plot in Figure 3a, we see that the oscillations in the $C_{D}\left(t\right)$ autocorrelation (see Figure 3c) correspond exactly to the observed large oscillations of the base pair displacements within the bubble region. These oscillations appear as a result of the rearrangement of the initial perturbation towards a more stable localised structure, which at this width (w=11 base pairs) takes a peak amplitude just below 2 Å, as can be seen in the 3D depiction of this stage of evolution in Figure 3e. The large oscillations are seen until times of a few tens of ps, whereafter the displacement profile exhibits a nearly constant-amplitude structure in the bubble region. The resulting, more stable bubble then slowly decays giving rise to the “slower” relaxation of the autocorrelation function visible in Figure 3c and earlier observed in Figure 2c until several nanoseconds. Then, the second stage of relaxation follows, which is characterised by a gradual spreading and the complete disappearance of the bubble, leading to complete equilibration (see Figure 3a), a process signified by the rapid decay of the autocorrelation function in the nanosecond to microsecond time scale (Figure 2c and Figure 3c).

The energy densities show a similar behaviour, but on a more muted scale as regards the initial oscillations during the bubble rearrangement at the first stage of relaxation. As demonstrated in Figure 3b, the initial oscillations are much smaller for the energy profile, and the rearranged localised structure remains almost stable for times up to nanoseconds. This is accompanied by a correspondingly flatter autocorrelation function (Figure 3d) for this period, before the complete thermalisation towards equilibrium. The 3D visualisation in Figure 3f shows the early oscillations followed by an almost constant-amplitude energy profile in the bubble region, with energy density per base pair just below 0.1 eV. In addition, in this case, the rapid decay of $C_{E}\left(t\right)$ at the second, final stage of the relaxation denotes the spreading and the gradual disappearance of the bubble (see Figure 3b,d).

These observations are consistent with the behavior depicted in Figure 2, namely that, for a fixed width, a greater amplitude of the initial bubble perturbation results in larger initial oscillations of the autocorrelation functions. Since these oscillations are produced by the rearrangement process mentioned above, the closer the initial bubble is to the nearly stable localised structure corresponding to its width, the smaller the changes required for the initial profile to be adapted to the inherent state. These smaller rearrangements are reflected as weaker oscillations in the autocorrelation functions, especially in the $C_{D}\left(t\right)$ functions where the oscillations are more evident.

It seems that both displacement and energy autocorrelation functions for the AT sequences in Figure 2a,b show the oscillatory region and the rapid decaying second stage, while the slow decaying relaxation behaviour is not evident, in contrast to what happens in the GC case. While not shown here, we have found that, in AT homopolymers, the inherent rearranged bubble has a dramatically shorter lifetime, corresponding to the lack of the “slow” relaxation region in this case (see Figure 2a,b). Because of this, the time required for a complete equilibration, signifying the loss of any memory about the initial perturbation, is around several nanoseconds for the AT homopolymer, which is orders of magnitude faster than the GC relaxation.

The decaying relaxation process depicted in these log-scale plots is very suggestive of a near stretched exponential behaviour. We thus consider a fit of the rapid decaying stage of the autocorrelation functions with a stretched exponential of the form

(10) $$C\left(t\right)=A\exp\left(-{\left(\frac{t}{\tau }\right)}^{\beta }\right)+\chi ,$$

where χ corresponds to the limiting value of the relevant autocorrelation function, while *A*, τ, and β are free parameters denoting the pre-exponential coefficient, the characteristic time constant, and the stretched exponent, respectively.

The dotted black lines in Figure 2b,d illustrate the stretched exponential fitted to the decay of the $C_{E}\left(t\right)$ data, where the fitting has started from the time where all data have entered the rapid decay relaxation stage (i.e., beyond the initial oscillations in the AT cases and the subsequent slow decay in the GC cases). This results in rather good fits, matching the data in all cases, and accurately capturing the decaying process. Critically, the good agreement with the data means that the fitted stretched exponential reaches the equilibrium value simultaneously with the measured data.

We note that the decay of the $C_{D}\left(t\right)$ data can also be fitted with stretched exponentials. However, the $C_{E}\left(t\right)$ curves provide a much more robust and consistent behaviour. Especially in the AT case, the $C_{D}\left(t\right)$ fits are very sensitive to the starting point of the fitting due to the relatively small decaying region after the oscillatory relaxation. Consequently, and since the time scales of both energy and displacement relaxations are similar (even visible in the autocorrelation functions themselves in Figure 2), we focus here on the relaxation timescales of the energy autocorrelation functions.

**Figure 4:**
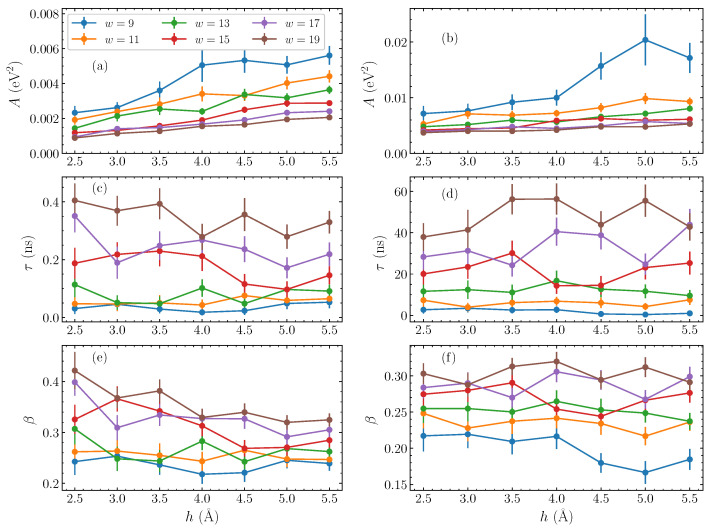
The stretched exponential parameters of Equation (10), as fitted to the energy autocorrelation functions $C_{E}\left(t\right)$ (Equation (8)), as functions of the amplitude *h*, for varying widths *w* (presented by different colors) of the initial bubble perturbation. The left column shows the parameters for AT sequences, and the right column for GC sequences. (**a**,**b**) the pre-exponential coefficient *A*; (**c**,**d**) the characteristic time constant τ; (**e**,**f**) the stretched exponent β. The line connections are used to guide the eye.

Having established this stretched exponential relaxation at relatively longer times, we now consider the dependence of the fitting parameters on the physical characteristics of the bubble perturbation—its amplitude *h* and width *w*. Performing similar fittings for a series of widths *w* between 9 and 19 base pairs, and taking amplitudes *h* between 2.5 and 5.5 Å, we find the corresponding fitting parameters of Equation (10) in each case. The obtained results are shown in Figure 4 as a function of the amplitude *h*, with the different widths represented as separate data sets. The parameters for AT homopolymers are shown in the left column, Figure 4a,c,e, and for the GC sequences on the right, Figure 4b,d,f.

Generally, the pre-exponential parameter *A* increases consistently with amplitude (Figure 4a,b), corresponding to the visible amplitude-dependence of the initial $C_{E}$ values, subtracting the corresponding $\chi_{E}$, seen in Figure 2b,d. When the width increases, however, *A* reduces, with the difference between the initial value of the autocorrelation function and its equilibrium value decreased.

Progressing through the parameters, we observe that the characteristic time τ (Figure 4c,d) is hardly affected by the amplitude of the initial bubble, in accordance with the results of Figure 2b,d. There are small, non-systematic variations with amplitude, but, in general, the parameters in Figure 4c,d remain around the same value on average as amplitude changes. The dependence on width on the other hand is very clear, with a steady decrease in τ values as the bubble width shrinks. This parameter most starkly reflects the difference in the relaxation dynamics between the AT and GC homopolymers. While the *A* (Figure 4a,b) and β (Figure 4e,f) parameters are of comparable size between the two homopolymers, the τ values in GC sequences are more than two orders of magnitude greater than their AT counterparts. This reflects a slower relaxation dynamics in the GC case. We note that, while the notion of a “large displacement” is not the same for AT and GC base pairs—the energy required to stretch an AT base pair to a certain displacement is much lower than the energy required to stretch a GC base pair to the same displacement—this discrepancy is certainly not on the scale of orders of magnitude.

The final fitting parameter, the stretched exponent β [Figure 4e,f], actually gently decreases with amplitude, implying that the shape of the relaxation does in fact change slightly with the bubble amplitude. However, the dependence of this parameter on the width *w* is clear, as in the case of τ; an increase of β is observed by increasing bubble widths.

We note that, in the autocorrelation function fittings, uncertainties are estimated using a bootstrapping approach on the $C_{E}\left(t\right)$ data points, sampling from the full set of runs. Consequently, the resultant errorbars in Figure 4 estimate the variance in the possible fits to the full data set.

The generally systematic behaviour of the fitting parameters suggests that there is a distinct trend in the behaviour of the overall relaxation dynamics as the amplitude and width of the initial bubble change. In order to understand this overall trend, we make use of the characteristic average time $\tau_{av}$ of the stretched exponential of Equation (10), which can be computed as [42,58,59]

(11) $$\tau_{av}=\frac{\Gamma \left(1/\beta \right)}{\beta }\tau ,$$

with Γ(x) being the conventional gamma function. This average time can be interpreted in this context as a measure of the relaxation time for the initial out-of-equilibrium perturbation, and provides an overall quantification of the time scale for the system’s memory of a bubble with particular amplitude *h* and width *w*. This quantity not only provides a relaxation time for the studied bubbles, but $\tau_{av}$ also enables us to use a single number as a descriptor for each autocorrelation function. Thus, in this sense, we reduce the complexity of many autocorrelation functions like those in Figure 2 to one number for each curve, enabling a much more effective comparison between relaxations for different initial bubbles.

**Figure 5:**
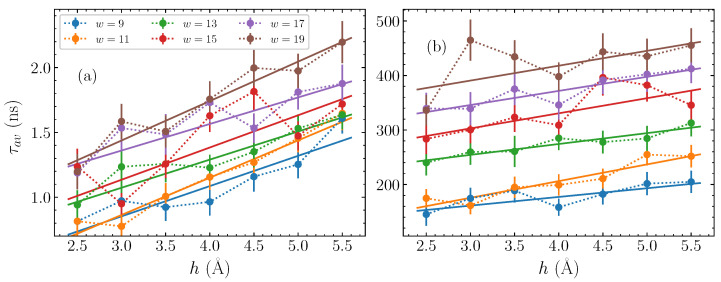
The average time $\tau_{av}$, Equation (11), of the stretched exponential fitted to the autocorrelation functions $C_{E}\left(t\right)$ (Equation (8)), obtained from the parameters shown in Figure 4, (**a**) for the AT sequences and (**b**) for the GC sequences (points). The dotted lines guide the eye for point-connection. The amplitude dependence of the average time is fitted by a linear function (see Equation (12)), depicted by solid lines in (**a**,**b**).

Based on the fitting parameters shown in Figure 4, the average time $\tau_{av}$, computed though Equation (11), is plotted in Figure 5, for each bubble width and amplitude considered here. The first panel, Figure 5a, shows $\tau_{av}$ against the bubble amplitude *h* for the AT homopolymers, with each different width represented by a differently coloured series of dotted-line-connected points. It is readily apparent that there is a consistent increase in the average relaxation time $\tau_{av}$ as the amplitude *h* grows, across all widths. Concerning the bubble width, there is a similarly clear indication that the wider bubbles have longer timescales, with the w=19 base pair bubbles (brown points in Figure 5a) having $\tau_{av}$ values larger than those seen for narrower w=9 base pair bubbles (blue points in Figure 5a), and a systematic change in between. The general relaxation timescales for the bubbles discussed here remain on the order of 0.5 to 2 ns.

Let us now consider the average times for the GC sequences, which are depicted in Figure 5b. Based on the observations of τ in Figure 4c,d, we expect a distinction between the relaxation time scales for the GC and AT sequences on the order of more than two orders of magnitude. This difference is clearly seen in comparing Figure 5a,b. Despite this difference in magnitudes, however, the same relative trends are visible that we saw in Figure 5a. More specifically, $\tau_{av}$ increases steadily with both amplitude and width. The w=19 base pair bubbles relax in more than twice the time than the w=9 base pair bubbles.

With the characterisation of the relaxation dynamics through the average time $\tau_{av}$, and the systematic behaviour exhibited by these average times in Figure 5, we are able to further quantify the effect of the bubble amplitude and width on the relaxation time. In particular, by fitting the data of Figure 5 with a straight line of the form,

(12) $$\tau_{av}=\tau_{0}+\alpha h,$$

for each width, we can find the lines of best fit for both the AT and GC cases, and estimate a value for the intercept $\tau_{0}$ and the slope α depending on the width *w*. These fits are shown by the solid lines in Figure 5, where in all cases the straight line provides an approximate description on average of the overall trend, thus reasonably capturing the dependence of $\tau_{av}$ on the bubble amplitude *h*. Consequently, for a fixed width *w*, given the parameters $\tau_{0}$ and α, it is possible to predict the typical relaxation time for a bubble of amplitude *h*.

**Figure 6:**
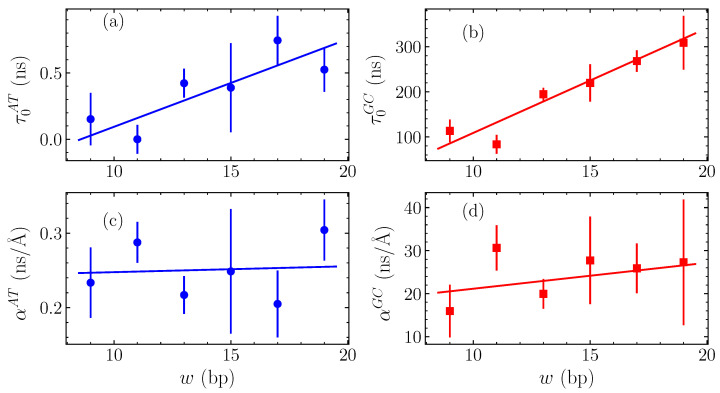
The variation of the parameters of linear fittings in $\tau_{av}$ (see the straight lines shown in Figure 5 and Equation (12)) with the width *w* measured in number of base pairs (bp). The vertical intercept $\tau_{0}$ is shown in (**a**) for AT sequences and in (**b**) for GC sequences, while the slope α is presented in (**c**) for AT homopolymers and in (**d**) for the GC ones (points). The data in all panels are in turn fitted with a straight lines (continuous lines).

The calculated fitting parameters $\tau_{0}$ and α are shown in Figure 6 for the GC and AT homopolymers, as functions of the bubble width *w*. In Figure 6a, we see the intercepts for the AT case, $\tau_{0}^{AT}$, while Figure 6b displays the GC intercepts $\tau_{0}^{GC}$. Both of these cases show a systematic approximately-linear increase of $\tau_{0}$ with *w*, corresponding to the apparently steady increase with width in the average relaxation time $\tau_{av}$ visible in Figure 5. The slopes $\alpha^{AT}$ and $\alpha^{GC}$ are given in Figure 6c,d for the AT and GC sequences, respectively, still as functions of the bubble width *w*. These values change significantly less with width than the intercepts, suggesting that the overall slope α is weakly dependent on *w*.

The variation of $\tau_{0}$ and α parameters depicted in Figure 6 enables us to complete the quantification of bubble relaxation times as a function of both the amplitude *h* and width *w* by fitting $\tau_{0}$ and α in turn with straight lines, shown as solid lines in all panels of Figure 6. For the intercepts $\tau_{0}$ in Figure 6a,b, a straight line was fitted finding $\tau_{0}^{GC}=23\left(5\right)w-120\left(70\right)$ ns for GC homopolymers and $\tau_{0}^{AT}=0.07\left(0.02\right)w-0.6\left(0.3\right)$ ns for AT. The numbers in parentheses denote the standard uncertainty in the last significant figure. For both homopolymers, this fit provides an accurate quantification of the dependence on *w*. The corresponding slopes α in Figure 6d,c are fitted by general linear fits, resulting in $\alpha^{GC}=0.6\left(0.9\right)w+20\left(10\right)$ ns/Å and $\alpha^{AT}=0.000\left(0.006\right)w+0.24\left(0.08\right)$ ns/Å for the GC and AT sequences, respectively, thus indicating a weak dependence on the bubble width.

Therefore, summarizing the results discussed above, we find that the characteristic bubble relaxation times of AT and GC homopolymers exhibit generally linear dependence on either bubble amplitude *h* or width *w*, described approximately through the relations

(13) $$\tau_{av}^{GC}=23w-120+\left(0.6w+20\right)h$$

(14) $$\tau_{av}^{AT}=0.07w-0.6+0.24h$$

where in these equations the bubble amplitudes are in Å, the widths in base pairs, and the average relaxation times in ns. The overall trend in the average relaxation times $\tau_{av}$ is the same for both AT and GC sequences, with the GC sequences relaxing around two orders of magnitudes more slowly than the AT sequences.

Bubble dynamics and, in particular, bubble closing and opening times have been considered in Ref. [60] within the framework of the Poland–Scheraga model. A Fokker–Planck equation for the probability density function to find a bubble containing *n* denatured base pairs at time *t* has been derived in the continuum limit. Through this formulation, it has been suggested that the closing time of a DNA bubble scales linearly with the bubble size [60]. This finding is in accordance with the indications of our results in Equations (13) and (14) that bubble relaxation times increase linearly with the bubble width *w*. Furthermore, the calculated relaxation times of less than microseconds in all cases considered here are consistent with previous detailed molecular dynamics simulations, finding that the short-time dynamics of DNA are only evident up to a few μs [7]. However, note that the latter computations concern equilibrium fluctuations, rather than relaxation of out-of-equilibrium perturbations, as considered here.

In a previous work within the same PBD framework, using identical parameters as here, apart from the fact that a common stacking parameter K=0.025 eV/Å${}^{2}$ was considered for both AT and GC sequences (which is close to the $K_{AA}=K_{TT}$ force constant used in this work, but more than 25% larger than the corresponding $K_{GG}=K_{CC}$ value), characteristic rates for the decay of local displacement and energy autocorrelation functions of equilibrium fluctuations were computed at various temperatures [42]. From the data presented in Figure 4 of that study, one can see that, at physiological temperatures, the characteristic times of base pair opening fluctuations are on the scale of tenths of ns for AT homopolymers and tens of ns for GC homopolymers, in accordance with the corresponding relaxation time scales obtained in the present work for the smaller widths. Furthermore, looking at the shape of the local autocorrelation functions for T close to physiological temperature in [42], weak oscillations are present in the timescale 1–10 ps, while the much faster decay in the case of AT as compared to the GC sequences is also very evident.

The substantial difference in bubble relaxation timescales between AT and GC homopolymers is a clear indication that the underlying base pairing dynamics play a strong role in determining the long-lasting effects of large out-of-equilibrium bubbles on the molecule. At thermal equilibrium, we have found that individual base pair opening fluctuations may on average live longer in the softer AT homopolymers as compared to the GC ones [54]. However, this result concerned bubbles of larger amplitude in the AT sequences than in the GC sequences, while both of these amplitudes were one order of magnitude smaller than the amplitudes considered here.

## 4. Conclusions

Within the framework of the Peyrard–Bishop–Dauxois model, we have computed the characteristic relaxation times for large bubbles in DNA homopolymers, using energy autocorrelation functions in order to study the equilibration of these coherent initial perturbations. Through simulations of up to a few microseconds, using efficient symplectic integration techniques, we ensure statistical accuracy of our results by averaging over many independent simulations (of the order of thousands). By varying the initial bubble amplitude and width in both pure AT and pure GC homopolymer DNA sequences, and computing autocorrelation functions for each case, we found that the decaying relaxation dynamics, after some initial oscillations, consistently develops according to a stretched exponential evolution, matching the complex temporal behavior of these autocorrelation functions (Figure 2). The mechanism of the whole relaxation dynamics is related to a two stage process where the initial bubble is first rearranged towards an inherent localized structure, and then this more stable structure eventually spreads and completely decays to equilibrium (Figure 3).

The autocorrelation functions have distinct average relaxation times, calculated through the parameters of the stretched exponential fittings (Figure 4) that depend on both bubble amplitude and width, with larger amplitude and wider bubbles exhibiting longer relaxation times. Computing the average relaxation times as a function of initial bubble amplitude *h* and width *w* enables the direct quantification of the dependence of $\tau_{av}$ on *h* and *w* through linear fittings (Figure 5 and Figure 6, Equations (13) and (14)).

The relaxation timescales for GC homopolymers are typically over two orders of magnitude longer than those evident in AT homopolymers; within the used model, GC relaxation times range between 150–500 ns, while the AT relaxations are on the order of 0.5–2 ns, for bubble amplitudes up to 5.5 Å and widths up to 19 base pairs that have been considered here. These findings demonstrate that large bubbles leave significant imprints on the long-term dynamics of DNA molecules, and the extent of this impact depends strongly on the base pair composition of the sequence.

A subsequent continuation of this work would be to study the effect of heterogeneity in the DNA sequence on the characteristic relaxation times. Furthermore, it would be interesting to investigate how these dynamics develop in functional gene promoter regions.

## Data Availability

The data supporting these results are available in the text and from the authors on reasonable request.

## References

[1] Beece D., Eisenstein L., Frauenfelder H., Good D., Marden M.C., Reinisch L., Reynolds A.H., Sorensen L.B., Yue K.T. (1980). Solvent viscosity and protein dynamics. Biochemistry.

[2] Ansari A., Berendzen J., Bowne S.F., Frauenfelder H., Iben I.E., Sauke T.B., Shyamsunder E., Young R.D. (1985). Protein states and proteinquakes. Proc. Natl. Acad. Sci. USA.

[3] Sobell H.M. (1985). Actinomycin and DNA Transcription. Proc. Natl. Acad. Sci. USA.

[4] Somoza M.M., Andreatta D., Murphy C.J., Coleman R.S., Berg M.A. (2004). Effect of lesions on the dynamics of DNA on the picosecond and nanosecond timescales using a polarity sensitive probe. Nucleic Acids Res..

[5] Pérez A., Javier Luque F., Orozco M. (2007). Dynamics of B-DNA on the microsecond time scale. J. Am. Chem. Soc..

[6] Banerjee D., Pal S.K. (2007). Direct Observation of Essential DNA Dynamics: Melting and Reformation of the DNA Minor Groove. J. Phys. Chem. B.

[7] Galindo-Murillo R., Roe D.R., Cheatham III T.E. (2014). On the absence of intrahelical DNA dynamics on the μs to ms timescale. Nat. Comm..

[8] Parmar J.J., Das D., Padinhateeri R. (2016). Theoretical estimates of exposure timescales of protein binding sites on DNA regulated by nucleosome kinetics. Nucleic Acids Res..

[9] Zeng Y., Zocchi G. (2006). Mismatches and bubbles in DNA. Biophys. J..

[10] Leroy J.L., Kochoyan M., Huynh-Dinh T., Guéron M. (1988). Characterization of base-pair opening in deoxynucleotide duplexes using catalyzed exchange of the imino proton. J. Mol. Biol..

[11] Jose D., Weitzel S.E., von Hippel P.H. (2012). Breathing fluctuations in position-specific DNA base pairs are involved in regulating helicase movement into the replication fork. Proc. Natl. Acad. Sci. USA.

[12] Phelps C., Lee W., Jose D., von Hippel P.H., Marcus A.H. (2013). Single-molecule FRET and linear dichroism studies of DNA breathing and helicase binding at replication fork junctions. Proc. Natl. Acad. Sci. USA.

[13] Lavery R., Zakrzewska K., Beveridge D., Bishop T.C., Case D.A., Cheatham T., Dixit S., Jayaram B., Lankas F., Laughton C. (2010). A systematic molecular dynamics study of nearest-neighbor effects on base pair and base pair step conformations and fluctuations in B-DNA. Nucleic Acids Res..

[14] Peyrard M., Cuesta-López S., James G. (2009). Nonlinear Analysis of the Dynamics of DNA Breathing. J. Biol. Phys..

[15] Manghi M., Destainville N. (2016). Physics of base-pairing dynamics in DNA. Phys. Rep..

[16] Hsu C.W., Fyta M., Lakatos G., Melchionna S., Kaxiras E. (2021). Ab initio determination of coarse-grained interactions in double-stranded DNA. J. Chem. Phys..

[17] Weber G., Haslam N., Whiteford N., Prugel-Bennett A., Essex J.W., Neylon C. (2006). Thermal equivalence of DNA duplexes without calculation of melting temperature. Nat. Phys..

[18] Zoli M. (2013). Helix untwisting and bubble formation in circular DNA. J. Chem. Phys..

[19] Freeman G.S., Hinckley D.M., Lequieu J.P., Whitmer J.K., de Pablo J.J. (2014). Coarse-grained modeling of DNA curvature. J. Chem. Phys..

[20] Zoli M. (2018). Twisting short dsDNA with applied tension. Physica A.

[21] Zoli M. (2018). End-to-end distance and contour length distribution functions of DNA helices. J. Chem. Phys..

[22] Wang X., Sun Z. (2019). Determination of Base-Flipping Free-Energy Landscapes from Nonequilibrium Stratification. J. Chem. Inf. Model..

[23] Peyrard M., Bishop A.R. (1989). Statistical Mechanics of a Nonlinear Model for DNA Denaturation. Phys. Rev. Lett..

[24] Dauxois T., Peyrard M., Bishop A.R. (1993). Entropy-driven DNA denaturation. Phys. Rev. E.

[25] Dauxois T., Peyrard M., Bishop A.R. (1993). Dynamics and Thermodynamics of a Nonlinear Model for DNA Denaturation. Phys. Rev. E.

[26] Dauxois T., Peyrard M. (1995). Entropy-driven transition in a one-dimensional system. Phys. Rev. E.

[27] Campa A., Giansanti A. (1998). Experimental tests of the Peyrard-Bishop model applied to the melting of very short DNA chains. Phys. Rev. E.

[28] Peyrard M., Farago J. (2000). Nonlinear localization in thermalized lattices: Application to DNA. Physica A.

[29] Kalosakas G., Ngai K.L., Flach S. (2005). Breather-induced anomalous charge diffusion. Phys. Rev. E.

[30] Kalosakas G. (2011). Charge transport in DNA: Dependence of diffusion coefficient on temperature and electron-phonon coupling constant. Phys. Rev. E.

[31] Chetverikov A.P., Ebeling W., Lakhno V.D., Velarde M.G. (2019). Discrete-breather-assisted charge transport along DNA-like molecular wires. Phys. Rev. E.

[32] Barré J., Dauxois T. (2001). Lyapunov exponents as a dynamical indicator of a phase transition. Europhys. Lett..

[33] Hillebrand M., Kalosakas G., Schwellnus A., Skokos C. (2019). Heterogeneity and chaos in the Peyrard–Bishop–Dauxois DNA model. Phys. Rev. E.

[34] Muniz M.I., Lackey H.H., Heemstra J.M., Weber G. (2020). DNA/TNA mesoscopic modeling of melting temperatures suggests weaker hydrogen bonding of CG than in DNA/RNA. Chem. Phys. Lett..

[35] Behnia S., Fathizadeh S., Javanshour S., Nemati F. (2020). Light-Driven Modulation of Electrical Current through DNA Sequences: Engineering of a Molecular Optical Switch. J. Phys. Chem. B.

[36] Alexandrov B.S., Gelev V., Monisova Y., Alexandrov L.B., Bishop A.R., Rasmussen K.Ø., Usheva A. (2009). A nonlinear dynamic model of DNA with a sequence-dependent stacking term. Nucleic Acids Res..

[37] Voulgarakis N.K., Kalosakas G., Rasmussen K.Ø., Bishop A.R. (2004). Temperature-Dependent Signatures of Coherent Vibrational Openings in DNA. Nano Lett..

[38] Ares S., Voulgarakis N.K., Rasmussen K.Ø., Bishop A.R. (2005). Bubble Nucleation and Cooperativity in DNA Melting. Phys. Rev. Lett..

[39] Ares S., Kalosakas G. (2007). Distribution of Bubble Lengths in DNA. Nano Lett..

[40] Kalosakas G., Ares S. (2009). Dependence on temperature and guanine-cytosine content of bubble length distributions in DNA. J. Chem. Phys..

[41] Tapia-Rojo R., Mazo J.J., Falo F. (2010). Thermal and mechanical properties of a DNA model with solvation barrier. Phys. Rev. E.

[42] Kalosakas G., Rasmussen K.Ø., Bishop A.R. (2006). Non-exponential decay of base-pair opening fluctuations in DNA. Chem. Phys. Lett..

[43] Choi C.H., Kalosakas G., Rasmussen K.Ø., Hiromura M., Bishop A.R., Usheva A. (2004). DNA dynamically directs its own transcription initiation. Nucleic Acids Res..

[44] Kalosakas G., Rasmussen K.Ø., Bishop A.R., Choi C.H., Usheva A. (2004). Sequence-specific thermal fluctuations identify start sites for DNA transcription. Europhys. Lett..

[45] Alexandrov B.S., Wille L.T., Rasmussen K.Ø., Bishop A.R., Blagoev K.B. (2006). Bubble statistics and dynamics in double-stranded DNA. Phys. Rev. E.

[46] Choi C.H., Rapti Z., Gelev V., Hacker M.R., Alexandrov B., Park E.J., Park J.S., Horikoshi N., Smerzi A., Rasmussen K.Ø. (2008). Profiling the thermodynamic softness of adenoviral promoters. Biophys. J..

[47] Alexandrov B.S., Gelev V., Yoo S.W., Bishop A.R., Rasmussen K.Ø., Usheva A. (2009). Toward a Detailed Description of the Thermally Induced Dynamics of the Core Promoter. PLoS Comput. Biol..

[48] Alexandrov B.S., Gelev V., Yoo S.W., Alexandrov L.B., Fukuyo Y., Bishop A.R., Rasmussen K.Ø., Usheva A. (2010). DNA dynamics play a role as a basal transcription factor in the positioning and regulation of gene transcription initiation. Nucleic Acids Res..

[49] Apostolaki A., Kalosakas G. (2011). Targets of DNA-binding proteins in bacterial promoter regions present enhanced probabilities for spontaneous thermal openings. Phys. Biol..

[50] Tapia-Rojo R., Prada-Gracia D., Mazo J.J., Falo F. (2012). Mesoscopic model for free-energy-landscape analysis of DNA sequences. Phys. Rev. E.

[51] Huang H.-H., Lindblad P. (2013). Wide-dynamic-range promoters engineered for cyanobacteria. J. Biol. Eng..

[52] Tapia-Rojo R., Mazo J.J., Hernandez J.A., Peleato M.L., Fillat M.F., Falo F. (2014). Mesoscopic Model and Free Energy Landscape for Protein-DNA Binding Sites: Analysis of Cyanobacterial Promoters. PLoS Comput. Biol..

[53] Hillebrand M., Kalosakas G., Bishop A.R., Skokos C. (2021). Bubble lifetimes in DNA gene promoters and their mutations affecting transcription. J. Chem. Phys..

[54] Hillebrand M., Kalosakas G., Skokos C., Bishop A.R. (2020). Distributions of bubble lifetimes and bubble lengths in DNA. Phys. Rev. E.

[55] Hairer E., Lubich C., Wanner G. (2002). Geometric Numerical Integration.

[56] Danieli C., Many Manda B., Mithun T., Skokos C. (2019). Computational efficiency of numerical integration methods for the tangent dynamics of many-body Hamiltonian systems in one and two spatial dimensions. Math. Eng..

[57] Blanes S., Moan P. (2002). Practical symplectic partitioned Runge–Kutta and Runge–Kutta-Nystróm methods. Journ. Comp. App. Math..

[58] Ngai K.L., Rendell R.W., Jain H. (1984). Anomalous isotope-mass effect in lithium borate glasses: Comparison with a unified relaxation model. Phys. Rev. B.

[59] Léon C., Luca M.L., Santamaria J. (1997). Correlated ion hopping in single-crystal yttria-stabilized zirconia. Phys. Rev. B.

[60] Hanke A., Metzler R. (2003). Bubble dynamics in DNA. J. Phys. A Math. Gen..

